# Prospective Attitudes towards Respiratory Syncytial Virus (RSV) Vaccination: Validation of a Survey Instrument among Young Females in Jordan Pending Vaccine Authorization

**DOI:** 10.3390/vaccines11081386

**Published:** 2023-08-19

**Authors:** Tleen Kherfan, Malik Sallam

**Affiliations:** 1 Department of Pathology, Microbiology and Forensic Medicine, School of Medicine, The University of Jordan, Amman 11942, Jordan; 2 Department of Clinical Laboratories and Forensic Medicine, Jordan University Hospital, Amman 11942, Jordan; 3 Department of Translational Medicine, Faculty of Medicine, Lund University, 22184 Malmö, Sweden

**Keywords:** vaccination perception, vaccine attitude assessment tool, vaccine rejection, respiratory virus, RSV, infants, pregnancy, Middle East, Jordan

## Abstract

In May 2023, the U.S. FDA advisors endorsed Pfizer’s pregnancy-administered vaccine (branded ABRYSVO) to protect infants from respiratory syncytial virus (RSV) infection. Vaccination can reduce the burden of RSV-related respiratory disease, with previous studies showing its substantial medical and financial burden in Jordan. However, pregnant women may exhibit hesitancy to get vaccinated due to concerns about potential risks to themselves or their fetuses. This study aimed to assess the acceptance of the RSV vaccine among young females and identify the determinants influencing their decision using a newly constructed instrument. A survey instrument was developed and validated, comprising 26 items to measure RSV vaccine acceptance. A cross-sectional study design was employed, with data collection from a sample of females aged 18 to 45 residing in Jordan during 5–6 July 2023, using a convenient approach via an online distributed questionnaire. The final study sample comprised 315 respondents, with 67.6% who have heard of RSV before the study. If the vaccine was safe, effective, and provided freely, 70.2% showed willingness to get the RSV vaccine, 15.2% resisted, and 14.6% were hesitant. Principal component analysis identified six internally consistent sub-scales with the following suggested themes: Advice, Burden, Conspiracy, Dangers, Efficiency, and Fear, comprising 21 items collectively as assigned as the “ABCDEF” scale for RSV vaccine acceptance. RSV vaccine acceptance in this study was associated with the advice and fear constructs. The validated survey instrument successfully captured important determinants of RSV vaccine acceptance among young females. RSV vaccine promotion efforts should focus on the following: enhancing vaccine education, improving trust in healthcare institutions and providers, reducing burdens through resolving cost issues and focusing on the role of social support, addressing safety concerns, and tailoring communication strategies to effectively promote the benefits of the vaccine. These insights can inform public health policies and interventions aiming to promote RSV vaccination and mitigate the burden of RSV-related diseases among infants. Follow-up studies are recommended with pregnant women as the target group to assess their attitude towards RSV vaccination and to confirm the validity of the conceived ABCDEF survey instrument.

## 1. Introduction

Respiratory syncytial virus (RSV) is a prominent causative agent for lower respiratory tract infections globally, particularly among infants and young children in low- and middle-income countries (LMICs) [1,2,3,4]. According to the Global Burden of Diseases, Injuries, and Risk Factors Study, RSV was implicated in 10.7 million cases of lower respiratory tract infections (LRTI) and over 41,000 deaths among children below the age of 5 years in the year 2016, despite the difficulty in obtaining accurate estimates [5,6]. The high burden of RSV infections is also manifested in its greater risk among infants compared to other respiratory viruses (e.g., influenza virus), highlighting the extensive burden of RSV on health care services and the need for effective preventive measures to reduce its burden [7,8,9,10]. In Jordan, previous studies showed the substantial burden of RSV disease in children in terms of severity, longer hospital stays, and financial costs [11,12].

The development of an effective and safe RSV vaccine was considered an utmost priority, based on the substantial burden of RSV disease, particularly among infants [13,14]. However, achieving this aim proved challenging in the past due to several reasons [15]. First, infants between the ages of 4 and 6 months may exhibit a compromised capacity to develop robust and durable adaptive memory responses subsequent to immunization [16,17]. Second, a significant hurdle in the development of RSV vaccine for infants lies in addressing the safety concern associated with vaccine-enhanced respiratory disease (ERD) [18]. This concern arises from the possibility of vaccinated children experiencing more severe respiratory symptoms upon subsequent infection with RSV [19]. Third, the narrow window of time between birth and the occurrence of the first RSV infection has posed a considerable obstacle to the direct vaccination of infants against RSV [4,15]. Maternal vaccination represents an underutilized strategy for protecting infants during the crucial and initial months of life. This approach involves the administration of vaccines to pregnant women, which leads to the transfer of protective antibodies to the developing fetus, passively conferring immunity to the newborns [20,21].

A vaccine targeting the RSV fusion (F) glycoprotein has shown promise in preventing severe RSV illness when administered to pregnant women, despite variability in efficacy [22,23,24,25]. However, challenges remain, such as determining the vaccine’s long-term effectiveness, assessing its safety profile, and optimizing its use in combination with other vaccines. Policy decisions are needed to address the implementation of maternal immunization and monoclonal antibody treatments in infants, while considering global health equity [26,27].

An interesting research area is the investigation of the willingness of pregnant women to get vaccinated [28]. Based on the extensive literature on the subject of vaccination hesitancy, several domains are expected to govern the decision of pregnant women to receive RSV vaccination [29,30,31]. Several relevant domains are expected to play the major role in the decision to get vaccinated, including (1) knowledge, attitude and beliefs about RSV and its vaccination; (2) perceived risks and concerns related to the vaccine and the disease in infants; (3) convenient access to vaccination and its prompt availability; (4) social influence and support; (5) personal experience of vaccination; (6) the belief in vaccine conspiracy; and (7) the calculation of decision-making to get vaccinated, among others [30].

To promote vaccine acceptance among pregnant women, it is important to address these domains comprehensively using validated methodologies [31]. Utilizing this approach is warranted to guide the customization of educational and communication vaccination campaigns, supported by evidence-based information [32,33,34]. Thus, the results can be used to tailor education and communication campaigns based on evidence-based information. Consequently, this approach could be helpful to address concerns and misconceptions, highlight the benefits of vaccination, and emphasize the safety and efficacy of the RSV vaccine during pregnancy.

Previous evidence showed the importance of employing a validated instrument as the mainstay measure to reach evidence-based conclusions necessary to guide intervention strategies to advocate vaccination [29,35]. Several survey instrument tools were previously utilized and culturally adapted to explore attitudes to vaccination in various population strata [36]. Example of these instruments include the Vaccine Hesitancy Scale [37,38], Vaccination Attitudes Examination Scale [39], Parent Attitudes About Childhood Vaccines [40,41], and the Vaccine Conspiracy Beliefs Scale [42]. The utility of these valid tools in exploring the determinants of attitude towards vaccination is invaluable for devising evidence-based intervention measures aiming to promote vaccination [30,43]. However, to the best of our knowledge, no survey instruments were previously conceived to examine the vaccination attitude among the specific sub-population of young women eligible for RSV vaccination to protect infants.

Therefore, the principal aim of the current study was the conceptualization and subsequent validation of a survey instrument tailored to assess RSV vaccine acceptance among women aged between 18 to 45 years, currently residing in Jordan.

The establishment of the validity of this instrument could facilitate comprehensive follow-up studies aimed at discerning the level of RSV vaccine acceptance and its contributory factors within the target group for RSV vaccination, namely pregnant women. Ultimately, this tool can facilitate future studies to address the factors associated with RSV vaccine acceptance among pregnant women, which is crucial for designing effective intervention strategies for vaccine promotion, achieving the aim of reducing RSV disease burden in infants.

The comprehensive National Immunization Programme in Jordan has been established since 1979 to ensure the availability, accessibility, and affordability of free vaccines to everyone in the country regardless of nationality [44]. Jordan has made significant strides in expanding its immunization coverage, particularly focusing on diseases of public health importance [45]. Despite these efforts, the country has encountered challenges related to vaccination hesitancy/rejection during the coronavirus disease 2019 (COVID-19) pandemic, which was reported at rates surpassing 60%, thus leading to suboptimal COVID-19 vaccine uptake [46,47,48,49]. Therefore, the study of attitude towards the prospective RSV vaccine, pending its introduction among the eligible population, holds particular importance given the vulnerability of infants to RSV-related disease and its high burden in the country [11,12].

## 2. Materials and Methods

### 2.1. Study Design

This study utilized a cross-sectional survey design to validate a survey instrument to assess the factors influencing RSV vaccine acceptance among females aged 18–45 years. The survey instrument was developed based on multiple domains known to impact women’s decision-making regarding vaccination, including knowledge, attitudes, beliefs, perceived risks and concerns, constraints hindering access to vaccination, social influence, personal vaccination experiences, and the decision-making process. These domains were identified through an extensive literature review that specifically examined the complex dimensions of vaccine hesitancy among pregnant women [50,51,52,53,54,55,56].

This study obtained approval by the Scientific Research Committee at the School of Medicine/University of Jordan (reference No. 3825/2023/67). Informed consent was obtained from all participants before their participation in the study. Participants were assured of the confidentiality and voluntary nature of their participation.

### 2.2. Eligibility Criteria and Sample Size Determination

Eligibility criteria included females currently residing in Jordan and aged between 18–45 years. The sample size was determined a priori based on the established guidelines for survey validation studies, considering the number of items (26) and 10 subjects per item yielding a minimum of 260 as the sample size [57,58]. The inclusion of women aged 18–45 years in this validation study is based on its relevance to the reproductive age group, which is critical for evaluating RSV vaccine acceptance among pregnant females in follow-up studies. By specifically targeting this age range, the study aimed to effectively capture the population relevant to assessing RSV vaccine acceptance, thereby informing future interventions and policies tailored to this specific target group.

### 2.3. Survey Instrument Development

The survey instrument was developed based on a comprehensive literature review and expert input [50,51,52,53,54,55,56]. The identified domains, namely knowledge, attitude and beliefs, perceived risks and concerns, access to vaccination, social influence, personal experience, and decision-making, and endorsement of vaccine conspiracies guided the construction of survey items, yielding a total of 26 items.

The content and face validity of the identified items were evaluated by two male doctors specializing in Laboratory Medicine. Their attributes included expertise in microbiology, immunology, and molecular diagnostics in addition to their competency in communication skills and diagnostic expertise. Additionally, the initial items were evaluated by two female medical technologists who were pregnant at the time, to ensure the survey coherence, simplicity, accuracy, and cultural appropriateness. The selection of the pregnant female medical technologists to assess the content and face validity of the initial survey items was justified by their background in healthcare and medical sciences, being detail-oriented as well as their personal experience that was helpful to evaluate pregnancy-related concerns.

### 2.4. Pilot Testing

Prior to questionnaire distribution, a pilot test was conducted face-to-face involving a sample of six females aged 27–41 years to refine the questionnaire. Feedback on item clarity, relevance, and appropriateness was obtained, leading to minor language modifications for improved clarity of the survey items. The results of the pilot test were excluded from the final sample.

### 2.5. Data Collection and Survey Content

The survey was created in the Arabic language on Google Forms. Then, the survey was distributed using the snowball sampling convenient approach starting by the contacts of the authors and encouraging the participants to share the survey link with their acquaintances. The following channels were used to distribute the survey: WhatsApp and Facebook messenger. The survey was distributed during 5–6 July 2023 and the decision to close the survey was based on reaching the minimum of 260+ valid responses. The selection of the snowball sampling approach using convenience sampling for this survey was justified by the need to reach a diverse and representative sample of participants due to limited resources and time constraints. The use of WhatsApp and Facebook messenger was justified based on their use as popular communication platforms in Jordan to facilitate the efficient distribution of the survey. The survey was created in the Arabic language to ensure the accessibility, increased response rate, and representativeness of different strata of society residing in Jordan.

Data were collected through the self-administered survey, which included an introductory section that provided a general overview on the study aims including a simplified details on the newly available vaccine for RSV disease prevention in infants, highlighting its efficacy and safety as demonstrated in clinical trials [22,59,60]. Additionally, clear instructions on how to complete the survey were provided besides the assurance of anonymity and confidentiality of the participants. This was followed by the consent item as follows: “Do you agree to participate in this study?” which was mandatory for completion of the survey.

The next section assessed the demographics of the participants: age (as a scale from 18 to 45 years), marital status (single, married, divorced, or widow), current number of children, educational level, work (student, unemployed, employed in healthcare fields, employed in non-healthcare-related fields), monthly income of household (≤1000 Jordanian dinar (JOD) vs. >1000 JOD), place of residence (the Capital Amman vs. outside the Capital), and nationality (Jordanian vs. non-Jordanian).

The knowledge of RSV was assessed at the beginning of the study with the item “Have you heard of RSV prior to this study?” using a binary response (yes/no), followed by the evaluation of 26 study-conceived items using a 5-point Likert scale (agree, somewhat agree, no opinion/I do not know, somewhat disagree, or disagree). These items were:

- RSV infection is considered dangerous among children;
- I believe that RSV vaccination for pregnant women will protect children from infection with the virus;
- I believe there are potential side effects of RSV vaccination for pregnant women;
- I think it is important for pregnant women to get RSV vaccination;
- I am confident in the safety and effectiveness of RSV vaccination for pregnant women;
- I would like to discuss RSV vaccination with healthcare providers before taking a position on it;
- I am concerned about possible side effects of RSV vaccination;
- I am afraid that vaccination against RSV during pregnancy may harm the fetus;
- I am concerned about the safety of vaccination in general for pregnant women;
- I have concerns about the long-term side effects of RSV vaccination on the health of pregnant women or the health of the fetus;
- The cost of RSV vaccination is an important factor in my attitude toward its acceptance;
- The cost of the RSV vaccination must be covered by the pregnant woman’s health insurance;
- I consider the healthcare providers’ recommendations important in shaping my opinion about RSV vaccination;
- I consider my husband’s support essential in shaping my decision to receive RSV vaccination during pregnancy;
- I consider the support of my family and social circle to be an important factor in shaping my decision to receive RSV vaccination during pregnancy;
- My previous experience with vaccinations has been generally positive;
- I would feel confident if the RSV vaccine was recommended during pregnancy by international organizations;
- I would feel confident if the RSV vaccine was recommended during pregnancy by the Ministry of Health;
- I prioritize my child’s health over any personal concerns I may have;
- The experiences and recommendations of other mothers will influence my attitude towards RSV vaccination during pregnancy;
- I would like more information about the benefits of RSV vaccination during pregnancy;
- I would like more information about the risks of RSV vaccination during pregnancy;
- Pharmaceutical companies that manufacture vaccines care about their financial gains at the expense of public health;
- Knowing more about the vaccine manufacturer is important for shaping my attitude and decision to get the vaccine;
- The expansion of vaccine manufacturing could be part of a global conspiracy to increase infertility and reduce human population; and
- The expansion of vaccine manufacturing could be part of a global conspiracy to increase abortions.

Then, the RSV vaccine acceptance was assessed using a 5-point Likert scale (agree, somewhat agree, no opinion/I do not know, somewhat disagree, or disagree) and the exact phrasing of the item was “I am willing to get the RSV vaccine during pregnancy if it is effective, safe, and is freely available”.

Responses that indicated potential carelessness were identified by examining answers to the following three items: “I am willing to pay an amount not exceeding 10 JOD for the RSV vaccine during pregnancy if it is effective and safe”, “I am willing to pay an amount not exceeding 100 JOD for the RSV vaccine during pregnancy if it is effective and safe”, and “I am willing to pay any amount of money for the RSV vaccine during pregnancy if it is effective and safe”. Inconsistent responses, such as expressing willingness to pay for the vaccine but unwillingness to receive it for free, or willingness to pay 100 JOD but unwillingness to pay 10 JOD, were considered as indications of careless responses, with subsequent exclusion from final analysis. Finally, to further assess careless responses, an image containing a number was included as an item in the survey, and the responses failing to accurately type the number were regarded careless. Completing all items was required for successful submission of the survey and this step was undertaken to eliminate the potential for item non-response.

### 2.6. Data Analysis

Descriptive statistics, including measures of central tendency (mean and median) and dispersion (standard deviation (SD) and interquartile range (IQR)), were employed to characterize the study variables. Associations between categorical variables were assessed using the chi-squared test (χ^2^). The non-parametric Mann–Whitney *U* (M-W) and Kruskal–Wallis (K-W) tests were utilized to evaluate the relationship between scale and categorical variables due to the non-normal distribution of the scale variables confirmed by the Kolmogorov–Smirnov (K-S) test. The internal consistency of the survey instrument constructs was examined using the Cronbach α coefficient. Construct validity was assessed through exploratory factor analysis (EFA) using principal component analysis (PCA). Oblimin rotation was applied to the extracted factors to account for potential correlation between factors. The suitability of the data for factor analysis was evaluated using the Kaiser–Meyer–Olkin (KMO) measure of sampling adequacy and Bartlett’s test of Sphericity. Statistical significance was determined at *p* < 0.050, and all analyses were conducted using IBM SPSS Statistics for Windows, Version 22.0. Armonk, NY, USA: IBM Corp.

## 3. Results

### 3.1. Participant Characteristics

**Figure 1:**
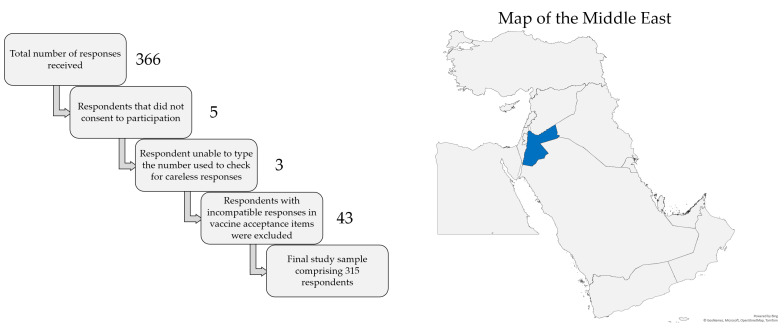
Representation of the filtration of careless responses to reach the final study sample (**left**) and the location of Jordan shown in blue color in the Middle East (**right**). The map was generated in Microsoft Excel, powered by Bing, © GeoNames, Microsoft, Navinfo, TomTom, Wikipedia. We are neutral with regard to jurisdictional claims in this map.

The total number of responses received was 366. Five respondents did not consent to participation, leaving a total of 361 respondents. Three additional responses were excluded due to the inability to type the number shown in the item used to check for careless responses, leaving a total of 358 respondents. Finally, 43 responses were excluded due to responses in vaccine acceptance items that did not appear compatible with each other (e.g., willingness to pay for the vaccine but unwillingness to get the vaccine for free). Thus, the final study sample comprised a total of 315 responses, as illustrated in Figure 1, which also shows a map of the location of Jordan in the Middle East.

Characteristics of the final study sample are illustrated in (Table 1).

### 3.2. Acceptability of RSV Vaccination in the Study Sample

A total of 221 participants either agreed, or agreed to some extent, with the statement “I am willing to get the RSV vaccine during pregnancy if it is effective, safe, and is freely available”, resulting in an overall RSV vaccine acceptance at a rate of 70.2%. Forty-eight participants disagreed with the aforementioned statement, at least to some extent, comprising the RSV vaccine resistance group (15.2%), while 46 had no opinion/did not know, thus forming the RSV vaccine hesitancy group (14.6%).

Stratified per demographic variables and the item that assessed previous knowledge of RSV, the resistance to receive a safe and effective RSV vaccine during pregnancy if provided freely was significantly more common among married participants and those having children (Table 2).

### 3.3. Psychometric Properties of the Developed Survey Instrument

The KMO measure of sampling adequacy yielded a value of 0.825, indicating that the dataset met the requirements for conducting factor analysis. Bartlett’s test of sphericity resulted in a highly significant *p* value of <0.001, providing evidence for the interrelatedness of the variables and justifying the utilization of factor analysis.

**Figure 2:**
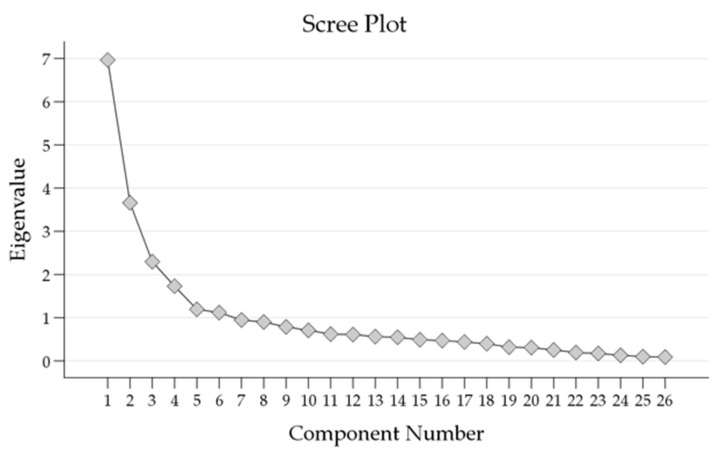
Scree plot of Eigenvalues showing the number of factors extracted from the Principal Component Analysis (PCA).

The scree plot (Figure 2) showed the eigenvalues of the components extracted through PCA.

The results of the PCA are presented in (Table 3), illustrating the initial eigenvalues, extraction sums of squared loadings, rotation sums of squared loadings for each component, and the cumulative percentage of variance explained.

The first six components displayed substantial eigenvalues, with the initial eigenvalue for Component 1 being 6.966. These components accounted for a cumulative percentage of variance of 65.2%. As subsequent components were added, the eigenvalues gradually decreased, indicating diminishing contributions to the overall variance.

The extraction sums of squared loadings and rotation sums of squared loadings closely mirrored the initial eigenvalues, highlighting the consistency of the findings. These results further support the significance of the first six components in explaining a substantial portion of the total variance. The PCA results suggested that retaining the first six components captures a significant portion of the variance.

The pattern matrix retrieved from the PCA with oblimin rotation and Kaiser normalization is shown in (Table 4).

The pattern matrix revealed distinct components capturing possible factors related to attitude to RSV vaccination as follows:

The first component appeared to be related to the importance placed on receiving recommendations from reputable sources, as well as the role of personal experiences in the attitude towards vaccination (items: 16, 17, and 18, loadings of −0.693 to −0.821). These items comprised the Advice sub-scale, which showed acceptable internal consistency with a Cronbach α value of 0.785.

The second component comprised items primarily highlighting the importance of reducing the perceived burden of vaccination based on the cost of vaccination and the role of partner, family, and social circle support of the decision to get vaccinated (items: 11, 14, and 15, loadings of 0.430 to 0.825). These items comprised the Burden sub-scale, which showed acceptable internal consistency with a Cronbach α value of 0.716.

The third component was related to specific conspiracy ideas regarding vaccination, comprising items 23, 25, and 26 (loadings ranging from 0.735 to 0.892). These items comprised the Conspiracy sub-scale, which showed good internal consistency with a Cronbach α value of 0.831.

The fourth component comprised four items related to perceived dangers and concerns from possible side effects of vaccination during pregnancy (items: 7, 8, 9, and 10, loadings ranging from 0.752 to 0.925). These items comprised the Danger sub-scale, which showed good internal consistency with a Cronbach α value of 0.888.

The fifth component, consisting of four items, was associated with the possible increased efficiency of interventions to increase vaccine acceptance through the following: covering the vaccine cost by health insurance, healthcare providers’ recommendations, and availability of information on vaccine safety and efficacy (items: 12, 13, 21, and 22, loadings ranging from 0.415 to 0.949). These items comprised the Efficiency sub-scale, which showed good internal consistency with a Cronbach α value of 0.841.

Finally, the sixth component, consisting of four items, was associated with the fear of RSV infection and its consequences in infants, and addressing this fear via vaccination (items: 1, 2, 4, and 5, loadings ranging from 0.752 to 0.925). These items comprised the Efficiency sub-scale, which showed acceptable internal consistency with a Cronbach α value of 0.745.

The combination of these six constructs resulted in inferring the “ABCDEF” scale for determination of the attitude towards RSV vaccination comprising 21 items.

### 3.4. ABCDEF Sub-Scale Correlation with RSV Vaccine Acceptance in the Study Sample

Assessment of the possible association between the developed ABCDEF sub-scales and the RSV vaccine acceptance in the whole study sample showed that the ABDEF sub-scales were significantly associated with RSV vaccine acceptance (Table 5). Conspiracy was the only sub-scale that did not show a statistically significant difference.

### 3.5. Multivariate Analysis Showed That Fear of RSV Disease in Infants and Advice from Credible Sources Were Independently Associated with RSV Vaccine Acceptance

Analyzing the association between the demographic variables with *p* < 0.100 in univariate analysis (having children and marital status), as well as the significant sub-scales (ABDEF) with RSV vaccine acceptance in the whole study sample, showed that the Advice and Fear constructs were significantly associated with RSV vaccine acceptance as opposed to resistance (aOR: 2.8, 95% CI: 1.2–6.4, *p* = 0.016 and aOR: 6.7, 95% CI: 2.4–18.5, *p* < 0.001, respectively, Table 6). On the other hand, fear was the only construct significantly associated with RSV vaccine acceptance vs. hesitancy (aOR: 4.5, 95% CI: 1.3–15.3, *p* = 0.016).

## 4. Discussion

On 3 May 2023, the U.S. Food and Drug Administration (FDA) granted approval to the first RSV vaccine in the world (AREXVY from GSK), specifically targeting individuals aged 60 and older, and it was approved by the European Union on 7 June 2023 [59,61]. The vaccine incorporates a stabilized form of the RSV F protein antigen [62]. Another RSV vaccine, (ABRYSVO from Pfizer), that is a bivalent recombinant subunit vaccine, was also approved by the FDA on 31 May 2023, for the same age group [59]. Additionally, a promising maternal RSV vaccine candidate, aimed at protecting infants up to 6 months of age, received favorable endorsement regarding its efficacy and safety from an FDA panel of advisors on 18 May 2023 [59].

The improved accessibility of RSV vaccines, among other preventive interventions, is of paramount importance, especially in LMICs where a significant fraction of respiratory disease-associated mortality, including deaths related to RSV infection in children occurs [63,64,65]. To fully harness the potential power of vaccination as a primary preventive approach against infectious diseases, it is crucial to carefully consider several pertinent factors. For example, the cost-effective vaccine distribution strategies are essential to reach vulnerable populations worldwide, besides the need to assess its sustained efficacy [66,67,68]. Importantly, the availability of vaccination against RSV by itself does not guarantee extracting its maximum benefits, thus highlighting the need for studies to determine the factors important for RSV acceptance and uptake [15,69,70]. The previous observations of reluctance to get vaccinated among pregnant women, primarily for safety concerns, further highlights the importance of addressing the determinants of vaccine acceptance among this target group [50,51,52,53,54,55,56].

Consequently, the purpose of this research was to devise and validate a comprehensive survey tool to explore the possible determinants of RSV vaccine acceptance among females of child-bearing age. The prominent underlying constructs that emerged from this investigation were Advice, Burden, Conspiracy, Dangers, Efficiency, and Fear, subsequently establishing the “ABCDEF” scale for assessment of possible factors associated with RSV vaccine acceptance.

The ABCDEF model effectively showed the multifaceted nature of RSV vaccination decision-making, which was highlighted previously by the notable work of different researchers worldwide [29,43,70,71,72,73]. The constructs inferred in the current study appear to play a major role in shaping the attitude to RSV acceptability and its adoption. The constructs provided an explanation of approximately 66% variance in attitude towards RSV vaccination, which can be crucial in discerning the nuanced aspects of willingness to receive this new vaccine.

In this study, the first inferred construct was termed “Advice”, comprising three items relating to individuals’ prior positive experiences with vaccination; their confidence if the RSV vaccine was recommended during pregnancy by national and international organizations. The “Advice” sub-scale emphasizes the key role that healthcare institutions, such as local ministries involved in health and welfare, as well as international organizations (e.g., the World Health Organization), play in serving as credible sources that enhance confidence in vaccination and shape individuals’ perceptions of vaccines [74,75]. Therefore, building confidence in a newly available vaccine is not only influenced by the content of communication messages but also by the source of advice, since it addresses the natural need for legitimacy in information conveyed [76]. This finding is consistent with previous evidence highlighting the significant role of trusted advice to shape attitude and beliefs towards vaccination [77,78]. Thus, the timely delivery of clear and consistent messaging from health authorities and practitioners to promote vaccination appears to be of prominent value [79]. Furthermore, the “Advice” construct underscores the significance of individuals’ previous positive experiences with vaccination, which can foster favorable attitudes and acceptance toward new vaccines if they are deemed necessary [80,81,82].

The second construct identified in this study, referred to as “Burden”, comprised three specific items. These items pertain to the influence of vaccine cost, spousal support, and support from family and social circles on individuals’ attitudes toward RSV vaccination during pregnancy. The construct of “Burden” sheds light on important aspects that can be addressed to alleviate challenges encountered during the implementation of vaccination among a vulnerable population, specifically for pregnant women. These burdens include the potential financial cost, which can serve as a significant barrier to accessing vaccination, particularly in LMICs [78,83]. Psychological burdens can be mitigated through the support of a partner, family members, and/or social circle, as their endorsement of the decision to receive the vaccine for the benefit of the infant can alleviate psychological concerns and foster RSV vaccine acceptance [84]. Addressing these burdens is crucial for optimizing the successful implementation of RSV vaccination among pregnant women, enhancing overall vaccine acceptance.

The third construct, termed the “Conspiracy” construct, provided insights into the impact of a few prevalent vaccine conspiracy theories on decision-making processes. This construct comprises three distinct items that show various aspects of these conspiratorial ideas. These include notions such as pharmaceutical companies prioritizing financial gains over public health, and the expansion of vaccine manufacturing being part of a global conspiracy to induce infertility, reduce the human population, and increase abortions. The pervasiveness of medical-related conspiracy beliefs poses a significant challenge to vaccination programs, necessitating proactive strategies for debunking misinformation [42,85,86,87]. Previous evidence showed that a stronger belief in vaccine conspiracy theories correlates with a lower rate of vaccine acceptance for various vaccines (e.g., COVID-19, influenza, and human papillomavirus vaccines, among others) [87,88,89,90]. Notably, this phenomenon holds particular importance in the Middle East and North Africa region, with Jordan serving as a salient example, where even well-informed groups like university students and health professionals demonstrated susceptibility to such beliefs [78,91,92].

The fourth construct identified in this validation study was termed “Dangers”. This construct explores the perceived risks and concerns associated with RSV vaccination. The construct included items that addressed potential side effects of RSV vaccination for pregnant women, concerns about possible side effects, fear of harm to the fetus due to vaccination during pregnancy, and general safety concerns regarding vaccination for pregnant women. The prominence of these constructs aligns with the extensive existing literature, which affirms the direct relationship between the perceived risk-benefit ratio and vaccine acceptance [93,94,95,96]. Higher perceived dangers are associated with lower likelihood of vaccine acceptance, which is consistent with the tenets of the Health Belief Model (HBM), which suggests less inclination to take health-related action when the potential risks outweigh the potential benefits [97,98]. The emergence of the “Dangers” construct highlights the importance of addressing vaccine safety concerns, in order to promote RSV vaccine acceptance during pregnancy [53,99].

The fifth construct identified in this study, termed the “Efficiency” construct, consisted of four specific items. These items addressed the significance of health insurance coverage for RSV vaccination during pregnancy, the impact of healthcare providers’ recommendations on individual perspectives regarding RSV vaccination, the interest in additional information concerning the benefits and risks of RSV vaccination during pregnancy, and the importance of communicating scientific evidence supporting the effectiveness of the RSV vaccine in reducing the risk in infants. Hence, enhancing the efficiency of vaccine promotion relies on the central role of healthcare providers in delivering essential information [100,101]. The belief in the vaccine’s efficacy plays a crucial role in the decision-making process, highlighting the critical importance of effective communication regarding vaccine benefits.

Lastly, the construct termed “Fear” involved four items. These items revolved around the dangers posed by RSV infection among infants, the belief that RSV vaccination for pregnant women offers protection against the virus for children, the importance placed on pregnant women receiving RSV vaccination, and the confidence in the safety and effectiveness of RSV vaccination for pregnant women. Additionally, maternal RSV immunization can prevent complications of RSV during pregnancy [102]. This construct highlighted the role of fear, which extends beyond concerns solely related to the vaccine itself, and included fear regarding the potential consequences of not getting vaccinated [103]. Therefore, maternal fear stemming from concerns about RSV disease and its potential severity can drive pregnant women to accept the vaccine as a preventive measure, aiming to protect their infants [104].

Another notable finding from the present validation study was the observed RSV vaccine acceptance rate of 70% among females of child-bearing age in the study sample. This finding suggested that a substantial proportion of individuals in this study have a favorable attitude to RSV vaccination during pregnancy, given that the vaccine is deemed safe, effective, and provided free-of-charge. This level of vaccine acceptance can be regarded as favorable, as it suggests that a significant number of individuals acknowledge the potential benefits of the vaccine in preventing RSV infection and its associated complications in infants. One potential explanation for this result in Jordan could be linked to the upsurge of RSV cases observed during the winter season of 2022/2023 among children in the country, as well as in other countries worldwide, which grasped considerable media attention [105,106,107]. This finding is aligned with the fact that 68% of the study participants reported having heard of RSV prior to the study. Another plausible explanation could be attributed to the introductory section of the questionnaire, which highlighted the efficacy and safety data derived from RSV vaccine trials among pregnant women, as well as the endorsement of the vaccine by the FDA advisors [59,60].

However, it is crucial to acknowledge that, despite the 70% acceptance rate for a safe, effective, and free RSV vaccine, a substantial portion of the sample still exhibited hesitancy or resistance towards receiving this vaccine. In the current study and through multinomial logistic regression, two specific constructs, namely “Advice” and “Fear”, emerged as relevant determinants of RSV vaccine acceptance as opposed to hesitancy or resistance. The “Advice” construct underscored the pivotal role played by credible healthcare institutions in shaping individuals’ attitudes towards RSV vaccination.

On the other hand, the “Fear” construct highlighted the potential impact of concerns related to the risks of RSV disease on infants as significant factors influencing the attitude towards RSV vaccination. Therefore, initial efforts aimed at promoting the benefits of RSV vaccination can benefit from effective communication that delivers clear and accurate information about the RSV vaccines. This communication should address individual concerns while emphasizing the safety data associated with the approved vaccines [22,59,60]. Additionally, messaging strategies can effectively convey the risks of RSV disease and the potential reduction in adverse outcomes, such as hospitalization and mortality, among infants, aiming to alleviate maternal fear, subsequently enhancing RSV vaccine uptake [108,109].

One of the strengths of the current study is the focus on validating a survey instrument specifically tailored to investigate the possible factors influencing RSV vaccine acceptance within a well-defined target population of females aged 18–45 years. Through the validation of this survey instrument for the specific demographic of women in their childbearing age, the current study could establish a scientific base for future research tackling the specific target population for RSV vaccination, namely pregnant women.

We encourage the utilization of the survey instrument in future studies to assess RSV vaccine acceptance among similar populations. Thus, the current study could contribute to a better understanding of the factors that could help to reduce the burden of RSV disease in infants through maternal vaccine promotion. Additionally, the study results can guide the development of targeted communication strategies and educational campaigns aimed at addressing specific determinants of maternal RSV vaccine acceptance through tailoring messaging to address concerns, misconceptions, and knowledge gaps identified in the study. Furthermore, conducting studies in different countries or regions can provide a broader perspective on the determinants of RSV vaccine acceptance given the global burden of RSV disease in infants.

Despite being the first to the best of our knowledge to tackle RSV vaccine acceptance pending final approval of the vaccine, several potential limitations should be considered: First, the utilization of a convenient sampling approach and an electronic survey format, could compromise the generalizability of the results due to selection bias with subsequent misrepresentation of the female population aged 18–45 years currently residing in Jordan. In future follow-up studies, the consideration of stratified random sampling is recommended to enhance the representativeness of the sample. The issue of reflexivity is pertinent as a study limitation since the survey distribution took place via the widely used online messaging platforms in Jordan. Although steps were taken to address this concern, such as analyzing survey content and seeking input from colleagues with expertise, it is important to acknowledge the potential impact of this factor when interpreting the study results, particularly considering the issue of possible misinformation spread through social media [110,111,112].

Second, the possibility of selection bias could be confounded, as it is plausible that individuals with a higher interest or knowledge regarding RSV may have been more inclined to participate in the survey. Consequently, this may have led to an overestimation of RSV knowledge and RSV vaccine acceptance rates, limiting the generalizability of the findings.

Third, it is important to recognize that participants in this study may have provided responses that they perceived as socially desirable, potentially deviating from their genuine beliefs or intentions regarding RSV vaccine acceptance. This introduces the potential for social desirability bias, which could have resulted in an overestimation of RSV vaccine acceptance rates and potentially compromised the accuracy of the findings.

Fourth, the adoption of a single-method (quantitative) approach in this study, driven by logistics and time constraints, limited the comprehensive assessment of determinants influencing RSV vaccine acceptance. Therefore, follow-up studies could benefit from incorporating qualitative interviews, which would offer deeper insights into the diverse perspectives within the study population, thereby enhancing the robustness of the conclusions.

Fifth, the current study focused solely on females currently residing in Jordan, coupled with the survey being available only in Arabic language. Consequently, the generalizability of the findings to a broader population is limited, considering that vaccine acceptance is context-specific and varies across different settings [30].

Sixth, the findings of this study were limited by the cross-sectional design, capturing data at a specific time point. This limitation restricts the understanding of potential changes in RSV vaccine acceptance over time, which is a crucial aspect considering that vaccine hesitancy is a dynamic phenomenon. Therefore, longitudinal studies are recommended to track changes in attitudes and behaviors related to RSV vaccine acceptance.

Finally, it is important to note that the effectiveness of vaccination programs is not solely dependent upon individual attitudes toward vaccination. It also encompasses a broader range of factors, including national policies, financial support, and various elements that contribute to both individual and collective perceptions of vaccination [113].

## 5. Conclusions

The “ABCDEF” scale developed and validated in this study included a wide range of determinants potentially influencing RSV vaccine acceptance among females of childbearing age. This scale could serve as a valuable tool for healthcare professionals and policymakers, offering a comprehensive framework to guide the development of targeted communication strategies aimed at promoting RSV vaccine uptake to reduce the disease burden among infants. Future research is recommended to gain a deeper understanding of the intricate decision-making processes surrounding maternal RSV vaccination. Subsequently, the accumulating evidence could optimize RSV vaccination outcomes.

## Figures and Tables

**Table 1:** The characteristics of the final study sample stratified based on whether the participant has heard of respiratory syncytial virus RSV or not (*n* = 315).

Variable	Category	Have You Heard of RSV before This Study?		*p* Value, χ^2^
		Yes N ^2^ (%)	No N (%)	
Age	≤30 years	86 (60.1)	57 (39.9)	0.010, 6.691
	>30 years	127 (73.8)	45 (26.2)	
Marital status	Single, divorced, or widow	67 (58.3)	48 (41.7)	0.007, 7.244
	Married	146 (73.0)	54 (27.0)	
Offspring	None	72 (57.6)	53 (42.4)	0.002, 9.501
	Having at least a child	141 (74.2)	49 (25.8)	
Current educational level	High school or less	20 (62.5)	12 (37.5)	0.062, 5.566
	Undergraduate	161 (65.7)	84 (34.3)	
	Postgraduate	32 (84.2)	6 (15.8)	
Employment status	Unemployed, students, or non-healthcare-related work	159 (63.9)	90 (36.1)	0.006, 7.688
	Healthcare-related work	54 (81.8)	12 (18.2)	
Monthly income of household	≤1000 JOD ^1^	147 (66.2)	75 (33.8)	0.441, 0.676
	>1000 JOD	66 (71.0)	27 (29.0)	
Residence	Amman (the Capital)	146 (69.2)	65 (30.8)	0.395, 0.724
	Outside the Capital	67 (64.4)	37 (35.6)	
Nationality	Jordanian	205 (69.7)	89 (30.3)	0.003, 8.957
	Non-Jordanian	8 (38.1)	13 (61.9)	

^1^ JOD: Jordanian dinar; ^2^ N: Number.

**Table 2:** Association between RSV vaccine acceptance and different study variables.

Variable	Category	RSV Vaccine Acceptance ^3^			*p* Value, χ^2^
		Acceptance Group N ^4^ (%)	Hesitancy Group N (%)	Resistance Group N (%)	
Age	≤30 years	106 (74.1)	20 (14.0)	17 (11.9)	0.275, 2.585
	>30 years	115 (66.9)	26 (15.1)	31 (18.0)	
Marital status	Single, divorced, or widow	81 (70.4)	23 (20.0)	11 (9.6)	0.024, 7.440
	Married	140 (70.0)	23 (11.5)	37 (18.5)	
Offspring	None	86 (68.8)	26 (20.8)	13 (10.4)	0.013, 8.687
	Having at least a child	135 (71.1)	20 (10.5)	35 (18.4)	
Current educational level	High school or less	21 (65.6)	4 (12.5)	7 (21.9)	0.434, 3.798
	Undergraduate	169 (69.0)	38 (15.5)	38 (15.5)	
	Postgraduate	31 (81.6)	4 (10.5)	3 (7.9)	
Employment status	Unemployed, students, or non-healthcare-related work	173 (69.5)	36 (14.5)	40 (16.1)	0.731, 0.628
	Healthcare-related work	48 (72.7)	10 (15.2)	8 (12.1)	
Monthly income of household	≤1000 JOD ^2^	153 (68.9)	37 (16.7)	32 (14.4)	0.260, 2.692
	>1000 JOD	68 (73.1)	9 (9.7)	16 (17.2)	
Residence	Amman (the Capital)	147 (69.7)	33 (15.6)	31 (14.7)	0.734, 0.617
	Outside the Capital	74 (71.2)	13 (12.5)	17 (16.3)	
Nationality	Jordanian	206 (70.1)	44 (15.0)	44 (15.0)	0.735, 0.617
	Non-Jordanian	15 (71.4)	2 (9.5)	4 (19.0)	
Have you heard of RSV ^1^ before this study?	Yes	147 (69.0)	30 (14.1)	36 (16.9)	0.487, 1.438
	No	74 (72.5)	16 (15.7)	12 (11.8)	

^1^ RSV: Respiratory syncytial virus; ^2^ JOD: Jordanian dinar; ^3^ RSV vaccine acceptance: Divided into three groups based on the response to the survey item “I am willing to get the RSV vaccine during pregnancy if it is effective, safe, and is freely available” which was assessed using a 5-point Likert scale, with those who agreed, or agreed to some extent, to the statement classified as the vaccine acceptance group, those who disagreed or disagreed to some extent to the statement were classified as the vaccine resistance group, while those who had no opinion or did not know were classified as the vaccine hesitancy group; ^4^ N: Number.

**Table 3:** The results of the Principal Component Analysis (PCA) showing the total variance explained by each component.

Component	Initial Eigenvalues			Rotation Sums of Squared Loadings
	Total	Percentage of Variance	Cumulative Percentage	Total
1	6.966	26.791	26.791	4.359
2	3.659	14.073	40.864	3.909
3	2.297	8.833	49.697	3.324
4	1.730	6.653	56.350	3.391
5	1.193	4.589	60.939	3.717
6	1.119	4.303	65.242	3.831
7	0.947	3.642	68.885	
8	0.900	3.463	72.348	
9	0.786	3.023	75.371	
10	0.707	2.720	78.091	
11	0.620	2.384	80.475	
12	0.611	2.349	82.824	
13	0.562	2.163	84.986	
14	0.547	2.102	87.089	
15	0.490	1.883	88.972	
16	0.471	1.811	90.783	
17	0.439	1.688	92.471	
18	0.397	1.528	93.999	
19	0.318	1.222	95.221	
20	0.308	1.185	96.406	
21	0.252	0.971	97.377	
22	0.193	0.744	98.121	
23	0.172	0.661	98.782	
24	0.130	0.500	99.282	
25	0.098	0.378	99.660	
26	0.088	0.340	100.00	

**Table 4:** Pattern matrix extracted from Principal Component Analysis (PCA) using oblimin rotation with Kaiser normalization.

N	Item	E	D	C	F	B	A
1	RSV infection is considered dangerous among children	0.019	−0.114	0.059	**−0.574**	0.040	0.134
2	I believe that RSV vaccination for pregnant women will protect children from infection with the virus	−0.027	0.102	−0.091	**−0.715**	−0.024	−0.213
3	I believe there are potential side effects of RSV vaccination for pregnant women	−0.158	**0.500**	0.230	0.269	0.254	0.002
4	I think it is important for pregnant women to get RSV vaccination	0.029	0.118	−0.058	**−0.687**	−0.053	−0.321
5	I am confident in the safety and effectiveness of RSV vaccination for pregnant women	−0.062	0.136	−0.161	**−0.557**	−0.037	−0.392
6	I would like to discuss RSV vaccination with healthcare providers before taking a position on it	**0.408**	−0.097	0.183	−0.369	0.117	0.067
7	I am concerned about possible side effects of RSV vaccination	−0.014	**0.752**	−0.006	0.153	−0.014	−0.055
8	I am afraid that vaccination against RSV during pregnancy may harm the fetus	0.007	**0.884**	0.004	−0.106	−0.008	0.036
9	I am concerned about the safety of vaccination in general for pregnant women	0.110	**0.925**	−0.029	−0.101	−0.112	0.075
10	I have concerns about the long-term side effects of RSV vaccination on the health of pregnant women or the health of the fetus	−0.010	**0.856**	0.029	−0.097	−0.020	0.011
11	The cost of RSV vaccination is an important factor in my attitude toward its acceptance	−0.142	−0.035	−0.364	−0.398	**0.430**	0.105
12	The cost of the RSV vaccination must be covered by the pregnant woman’s health insurance	**0.436**	−0.063	0.106	−0.385	0.171	−0.073
13	I consider the healthcare providers’ recommendations important in shaping my opinion about RSV vaccination	**0.415**	−0.031	0.144	−0.268	0.398	−0.027
14	I consider my husband’s support essential in shaping my decision to receive RSV vaccination during pregnancy	0.090	−0.101	0.018	0.051	**0.825**	−0.088
15	I consider the support of my family and social circle to be an important factor in shaping my decision to receive RSV vaccination during pregnancy	0.039	−0.040	−0.054	0.011	**0.804**	−0.112
16	My previous experience with vaccinations has been generally positive	0.066	−0.050	0.048	−0.002	−0.099	**−0.693**
17	I would feel confident if the RSV vaccine was recommended during pregnancy by international organizations	0.066	−0.034	0.033	−0.030	0.123	**−0.821**
18	I would feel confident if the RSV vaccine was recommended during pregnancy by the Ministry of Health	−0.027	0.040	0.053	−0.071	0.212	**−0.805**
19	I prioritize my child’s health over any personal concerns I may have	**0.404**	−0.113	−0.087	0.020	0.176	−0.309
20	The experiences and recommendations of other mothers will influence my attitude towards RSV vaccination during pregnancy	0.014	−0.124	−0.370	−0.075	0.218	−0.258
21	I would like more information about the benefits of RSV vaccination during pregnancy	**0.923**	0.052	−0.072	0.054	−0.003	−0.050
22	I would like more information about the risks of RSV vaccination during pregnancy	**0.949**	−0.013	−0.046	0.083	−0.064	−0.049
23	Pharmaceutical companies that manufacture vaccines care about their financial gains at the expense of public health	−0.143	−0.067	**0.735**	0.013	0.060	0.118
24	Knowing more about the vaccine manufacturer is important for shaping my attitude and decision to get the vaccine	**0.401**	0.051	−0.345	0.140	0.349	−0.025
25	The expansion of vaccine manufacturing could be part of a global conspiracy to increase infertility and reduce human population	0.016	0.068	**0.892**	−0.010	−0.019	−0.129
26	The expansion of vaccine manufacturing could be part of a global conspiracy to increase abortions	0.072	0.126	**0.880**	−0.014	−0.032	−0.137

Extraction method: principal component analysis, rotation method: oblimin with Kaiser normalization, the rotation converged in 11 iterations, RSV: respiratory syncytial virus, A: advice, B: burden, C: conspiracy, D: danger, E: efficiency, F: fear. A cut-off value of 0.400 in the pattern matrix was used as the threshold to determine the significance of loadings (highlighted in bold).

**Table 5:** Sub-scale correlation with the RSV vaccine acceptance.

RSV ^1^ Vaccine Attitude Group ^2^	Mean ± SD ^9^, Median (IQR ^10^)
**Advice sub-scale ^3^**	
Acceptance	12.4 ± 2.3, 13.0 (11.0–15.0)
Hesitancy	10.6 ± 2.4, 10.0 (9.0–12.0)
Resistance	9.5 ± 3.4, 9.0 (7.0–12.0)
Kruskal–Wallis H, *p* value	44.946, <0.001
**Burden sub-scale ^4^**	
Acceptance	12.2 ± 2.6, 12.0 (11.0–14.0)
Hesitancy	10.9 ± 2.2, 11.0 (9.0–12.0)
Resistance	10.9 ± 3.2, 11.0 (8.3–13.0)
Kruskal–Wallis H, *p* value	18.768, <0.001
**Conspiracy sub-scale ^5^**	
Acceptance	7.0 ± 3.2, 6.0 (4.0–9.0)
Hesitancy	7.7 ± 2.5, 8.5 (6.0–9.0)
Resistance	6.9 ± 3.1, 6.0 (5.0–9.0)
Kruskal–Wallis H, *p* value	3.002, 0.223
**Danger sub-scale ^6^**	
Acceptance	8.2 ± 3.4, 8.0 (5.0–10.5)
Hesitancy	8.2 ± 2.9, 8.0 (6.0–11.0)
Resistance	6.9 ± 3.3, 5.5 (4.0–8.8)
Kruskal–Wallis H, *p* value	8.023, 0.018
**Efficiency sub-scale ^7^**	
Acceptance	18.1 ± 2.3, 19.0 (17.0–20.0)
Hesitancy	16.1 ± 2.8, 16.0 (14.0–19.0)
Resistance	16.2 ± 4.0, 18.0 (13.0–20.0)
Kruskal–Wallis H, *p* value	26.355, <0.001
**Fear sub-scale ^8^**	
Acceptance	16.4 ± 2.6, 17.0 (14.0–18.0)
Hesitancy	14.7 ± 2.9, 14.0 (13.0–17.0)
Resistance	12.9 ± 2.9, 12.5 (11.0–15.0)
Kruskal–Wallis H, *p* value	53.050, <0.001

^1^ RSV: Respiratory syncytial virus; ^2^ Vaccine attitude group was divided into three groups based on the response to the survey item “I am willing to get the RSV vaccine during pregnancy if it is effective, safe, and is freely available”, which was assessed using a 5-point Likert scale with those who agreed, or agreed to some extent, to the statement classified as the vaccine acceptance group, those who disagreed or disagreed to some extent to the statement were classified as the vaccine resistance group, while those who had no opinion or did not know were classified as the vaccine hesitancy group; ^3^ Advice sub-scale: higher scores indicate higher agreement with the role of personal experience and confidence in the advice of credible sources recommending RSV vaccination; ^4^ Burden sub-scale: higher scores indicate higher agreement with the role of vaccine cost and partner, family, and social circle support in determining attitude to RSV vaccination; ^5^ Conspiracy sub-scale: lower scores indicate higher agreement conspiratorial statements; ^6^ Danger sub-scale: higher scores indicate lower perceived danger related to RSV vaccine side effects; ^7^ Efficiency sub-scale: higher scores indicate higher agreement with the potential efficiency of RSV vaccine acceptance if the vaccine cost is covered by health insurance, healthcare providers’ recommendations, and availability of information on vaccine safety and efficacy; ^8^ Fear sub-scale: higher scores indicate higher agreement with fear of RSV disease in infants and belief in RSV vaccine efficacy and its importance for infant protection; ^9^ SD: Standard deviation; ^10^ IQR: Interquartile range.

**Table 6:** Multinomial logistic regression showing the association between the ABCDEF sub-scales with RSV vaccine acceptance.

**RSV ^1^ Vaccine Acceptance vs. RSV Vaccine Resistance ^2^**	**aOR ^8^ 95% CI ^9^**	***p* Value**
Advice score ^3^ >12 vs. ≤12	2.791 (1.219–6.388)	0.015
Burden score ^4^ >12 vs. ≤12	1.050 (0.467–2.361)	0.907
Danger score ^5^ >8 vs. ≤8	2.045 (0.947–4.420)	0.069
Efficiency score ^6^ >19 vs. ≤19	1.135 (0.517–2.492)	0.752
Fear score ^7^ >16 vs. ≤16	6.720 (2.441–18.497)	<0.001
Marital status: Single, divorced, or widow vs. married	1.695 (0.477–6.028)	0.415
Offspring: None vs. having at least a child	0.871 (0.259–2.928)	0.823
**RSV vaccine acceptance vs. RSV vaccine hesitancy ^3^**		
Advice score >12 vs. ≤12	1.183 (0.397–3.521)	0.763
Burden score >12 vs. ≤12	0.337 (0.108–1.049)	0.060
Danger score >8 vs. ≤8	1.932 (0.767–4.869)	0.162
Efficiency score >19 vs. ≤19	0.595 (0.206–1.721)	0.338
Fear score >16 vs. ≤16	4.488 (1.319–15.270)	0.016
Marital status: Single, divorced, or widow vs. married	1.822 (0.421–7.883)	0.422
Offspring: None vs. having at least a child	2.147 (0.521–8.849)	0.290

^1^ RSV: Respiratory syncytial virus; ^2^ Vaccine attitude groups were divided into three groups based on the response to the survey item “I am willing to get the RSV vaccine during pregnancy if it is effective, safe, and is freely available”, which was assessed using a 5-point Likert scale, with those who agreed, or agreed to some extent, to the statement classified as the vaccine acceptance group, those who disagreed or disagreed to some extent to the statement were classified as the vaccine resistance group, while those who had no opinion or did not know were classified as the vaccine hesitancy group; ^3^ Advice sub-scale: higher scores indicate higher agreement with the role of personal experience and confidence in the advice of credible sources recommending RSV vaccination; ^4^ Burden sub-scale: higher scores indicate higher agreement with the role of vaccine cost and partner, family, and social circle support in determining attitude to RSV vaccination; ^5^ Danger sub-scale: higher scores indicate lower perceived danger related to RSV vaccine side effects; ^6^ Efficiency sub-scale: higher scores indicate higher agreement with the potential efficiency of RSV vaccine acceptance if the vaccine cost is covered by health insurance, healthcare providers’ recommendations, and availability of information on vaccine safety and efficacy; ^7^ Fear sub-scale: higher scores indicate higher agreement with fear of RSV disease in infants and belief in RSV vaccine efficacy and its importance for infant protection; ^8^ aOR: Adjusted odds ratio; ^9^ CI: Confidence interval.

## Data Availability

The data presented in this study are available on request from the corresponding author (M.S.).

## Abbreviations

ABCDEF	Advice, Burden, Conspiracy, Dangers, Efficiency, and Fear
aOR	Adjusted odds ratio
CI	Confidence interval
COVID-19	Coronavirus disease 2019
EFA	Exploratory factor analysis
ERD	Vaccine-enhanced respiratory disease
F	RSV fusion glycoprotein
FDA	The United States Food and Drug Administration
HBM	Health Belief Model
IQR	Interquartile range
JOD	Jordanian dinar
KMO	Kaiser-Meyer-Olkin measure
K-S	Kolmogorov-Smirnov test
K-W	Kruskal-Wallis test
LMICs	Low- and middle-income countries
LRTI	Lower respiratory tract infection
M-W	Mann-Whitney U test
PCA	Principal component analysis
RSV	Respiratory syncytial virus
SD	Standard deviation
